# LTK-YOLO: Lightweight Keypoint Detection for Robotic Tea Bud Picking

**DOI:** 10.3390/plants15203178

**Published:** 2026-10-16

**Authors:** Xiaomei Yi, Zekai Zheng, Hao Liang, Yifan Huo, Ming Liu, Lili He

**Affiliations:** 1 College of Computer Science and Technology (College of Artificial Intelligence), Zhejiang Sci-Tech University, Hangzhou 310018, China; 2 College of Artificial Intelligence, Zhejiang A&F University, Hangzhou 311300, China

**Keywords:** tea bud detection, picking point detection, lightweight model, LTK-YOLO, Euclidean distance

## Abstract

In unstructured tea garden environments, the high color similarity between tea buds and surrounding leaves, together with severe clustering and occlusion, significantly reduces the localization accuracy of picking points and limits the operational efficiency of tea-picking robots. Existing studies often improve detection performance by increasing network depth, but overlook the deployment constraints of resource-limited edge devices. To address these challenges, this study proposes a lightweight end-to-end tea bud picking point detection model based on YOLOv8-Pose, named LTK-YOLO. The proposed model integrates object detection and keypoint localization within a unified framework. First, a WTConv operation is embedded into the bottleneck of the C2f module to construct a C2f-WTConv structure, enabling wavelet-domain feature extraction that enhances contextual representation while reducing redundant computation and memory access. Second, the adaptive upsampling operator DySample replaces conventional interpolation-based upsampling in the feature fusion stage, improving multi-scale feature reconstruction through learnable point sampling. Third, a weighted Shape-NWD loss function is introduced to incorporate bounding box shape and scale information, which accelerates convergence and improves keypoint localization accuracy for small and occluded targets. Experiments were conducted on a self-built tea bud dataset containing 4000 images, divided into training, validation, and test sets at a ratio of 8:1:1. The proposed LTK-YOLO achieved an F1 score of 91.95% and a ${\mathrm{mAP}}_{kp50}$ of 92.87%, with an average Euclidean localization error below 15 pixels. Compared with the baseline YOLOv8s-Pose, LTK-YOLO improved ${\mathrm{AP}}_{kp50}$ by 3.56 and 4.02 percentage points for kp1-1 and kp1-2, respectively, reduced Params by 53.10%, and GFLOPs by 32.75%. In addition, the model achieved an inference speed of 85.86 FPS on an NVIDIA RTX 4060 GPU, demonstrating strong real-time capability. Because the dataset was captured in a single spring season at two tea gardens in Hangzhou, the reported validation results are bounded to comparable tea varieties, canopy density, and illumination and occlusion conditions; deployment in other climatic regions, seasons, or on unseen tea varieties is expected to require fine-tuning with locally collected data. Overall, LTK-YOLO achieves a favorable balance between detection accuracy, computational efficiency, and lightweight design, providing a promising solution for real-time tea-picking robotic systems.

## 1. Introduction

With the sustained growth of global tea demand and the continued expansion of planting areas, tea production is accelerating its transformation toward mechanization and intelligence. According to FAO statistics, the global tea-harvested area reached approximately 4.7 million hectares in 2023, of which China accounted for about 2.95 million hectares, ranking first worldwide [1]. Premium teas are harvested to strict agronomic standards that typically accept only tender “one bud and one leaf” or “one bud and two leaves” shoots, and the picking point must lie within a narrow segment of the stem rather than at any visually salient location. Tea bud picking is therefore one of the most critical links in the tea production process, because the timing and location of the cut directly determine the quality grade and economic value of the picked tea [2]. Traditional manual picking is labor-intensive, inefficient, and heavily dependent on worker experience, which makes it difficult to meet the demands of large-scale, high-quality tea production [3]. Accurate identification of tea buds and reliable picking point localization in unstructured tea garden environments have thus become key obstacles to the adoption of intelligent tea-picking equipment [4].

Early research on tea bud detection relied primarily on traditional machine vision methods that used hand-crafted color, texture, and morphological features for target segmentation and recognition [5]. For example, ref. [6] proposed a tea bud segmentation method based on an improved watershed algorithm that extracts tea bud regions through multi-channel color feature analysis, whereas [7] combined simple linear iterative clustering (SLIC) with a support vector machine (SVM) for tea disease and target recognition in complex backgrounds. Although these methods achieved promising results under controlled conditions, their feature design depends heavily on domain experience and is highly sensitive to changes in lighting, foliage occlusion, and background interference, which limits their detection stability and generalization in unstructured real tea gardens [8].

With advances in deep learning, convolutional neural networks (CNNs) have been widely applied to agricultural perception tasks, including fruit picking location [9], disease detection [10], yield prediction [11], and maturity recognition [12]. Compared with traditional methods, CNN-based models automatically learn high-level semantic representations, significantly improving adaptability to complex tea garden environments [13]. Mainstream CNN detectors are generally divided into two-stage and single-stage frameworks.

Two-stage detectors, such as Faster R-CNN [14] and Mask R-CNN [15], first generate region proposals and then perform classification and regression. Ref. [16] combined Faster R-CNN with a fully convolutional network (FCN) to segment the tender stems of tea buds and localize picking points, reporting an accuracy of 84.91% and an inference speed of 52.9 FPS, while [17] improved Mask R-CNN to build the MR3P-TS model, which attained a picking point localization accuracy of 94.9% and an inference speed of 49.8 FPS. However, the two-stage pipeline processes region proposal generation and target classification or regression sequentially [18]; its complex architecture and high computational cost make it difficult to meet the real-time requirements of intelligent tea-picking equipment.

In contrast, single-stage detectors have been increasingly adopted for tea bud detection and picking point localization. Models such as YOLO [19] and SSD [20] directly predict object categories and bounding boxes in a unified pipeline. Ref. [21] combined an improved YOLOX-tiny with PSP-Net to perform tea bud region detection and tea stem segmentation simultaneously. Ref. [22] carried out tea bud detection with an improved YOLOv4 network and combined HSV color segmentation with geometric analysis to compute picking points. Ref. [23] proposed the lightweight network MC-DM (integrating DeepLabV3+ and MobileNetV2), which improved tea bud segmentation and picking point localization in natural environments. However, these methods usually rely on additional geometric modeling or post-processing steps, and their robustness and generalization remain limited in unstructured tea garden scenes with severe occlusion or large variations in target scale [24].

With the recent development of pose estimation and keypoint detection, keypoint-based target detection has become a growing research focus. For example, ref. [25] proposed YOLOv8PoseBoost, which achieves high-precision detection of small-target picking points in complex scenes. Ref. [26] proposed YOLOv9-pose and YOLOv10-pose and verified their comprehensive advantages in target detection and keypoint localization. Ref. [27] introduced keypoint detection into the picking point localization of cherry tomatoes, proposing the YOLO-PP network, and achieved accurate identification of stem keypoints. More recently, ref. [28] proposed an improved YOLOv7-based network for tea bud pose estimation and grading detection, further demonstrating the potential of pose-based methods in tea harvesting. Although the YOLO-Pose family performs well in target detection and keypoint localization, its multi-scale feature fusion usually relies on a pyramid structure, which can lose fine-grained feature information. In tea garden scenes with densely distributed small targets and severe occlusion, detection performance therefore still leaves room for improvement [29].

Meanwhile, lightweight network design has become a major trend for deploying detection models on resource-constrained agricultural edge devices. General-purpose lightweight architectures such as MobileNetV2 [30], EfficientNet [31], and GhostNet [32] reduce parameters and computation through depthwise separable convolutions, compound scaling, and cheap feature generation, respectively, and have been widely adopted as backbones for in-field perception. In tea garden scenarios specifically, ref. [33] integrated GhostNet with YOLOv5 for lightweight tea bud recognition; ref. [34] proposed an improved lightweight YOLOv5 for tea bud identification and localization; ref. [35] developed a MobileNetV3-based lightweight tea bud grading detection model for embedded applications; and ref. [36] proposed GLS-YOLO with a GhostNetV2 backbone for lightweight tea bud detection in complex scenarios. However, these studies mainly address bud detection or grading, and few tackle keypoint-level picking point localization within a unified lightweight framework.

In summary, although existing research on tea bud detection and picking point location has made some progress, it still faces the following challenges: (1) The unstructured tea garden environment is complex, and the model needs to ensure high detection accuracy while taking into account lightweight and real-time performance; (2) The multi-stage detection and post-processing process is redundant, which reduces the overall positioning efficiency; (3) The tea bud target size is small and the pixel ratio is low, which limits the feature expression ability; (4) The overlapping of branches and leaves, uneven lighting and significant differences in varieties affect the robustness and generalization performance of the model.

To address the above challenges, this paper proposes a lightweight tea bud keypoint detection model, LTK-YOLO, based on YOLOv8s-Pose. The main contributions are summarized as follows:

(1) A tea bud picking point detection dataset containing 4000 images across four premium tea varieties was constructed, together with an agronomic annotation scheme that encodes the national harvesting standards for “one bud and one leaf” and “one bud and two leaves” picking points, providing a reusable benchmark for tea bud localization research.

(2) Beyond a plain combination of existing operators, we systematically identify where wavelet-domain convolution benefits the C2f structure—embedding WTConv in the bottleneck outperforms shallow- and mid-level placements (Section 2.2)—and we parameterize the weighted Shape-NWD loss by the scale statistics of tea bud bounding boxes in the dataset. Integrating these components with the DySample adaptive upsampling operator into YOLOv8s-Pose yields the lightweight tea bud keypoint detection model LTK-YOLO. Compared to the baseline model, this model achieves ${\mathrm{AP}}_{kp50}$ gains of 3.56 and 4.02 percentage points for kp1-1 and kp1-2, respectively, and reduces Params and GFLOPs by 53.10% and 32.75%, respectively, achieving a better balance between accuracy and efficiency.

(3) In the task of detecting tea bud picking points, we compared the performance of LTK-YOLO with other mainstream keypoint detection models. The verification results show that the model achieves the lowest average Euclidean localization error and the smallest error fluctuation, ensuring reliable localization of picking points even under unstructured tea garden conditions.

## 2. Results

### 2.1. Experimental Setup and Evaluation Indicators

All experiments were conducted using the PyTorch deep learning framework, with YOLOv8s-Pose serving as the baseline model. The experimental platform consisted of a Windows 11 operating system, an Intel Core i7-13700HX CPU, an NVIDIA RTX 4060 GPU, and 32 GB of RAM. The software environment included Python 3.8.2, CUDA 11.7, and PyTorch 2.0.0, configured via Anaconda 3 and PyCharm 2026.2.1. To ensure reproducibility, the random seed was fixed at 42 for Python, NumPy 2.4.0, and PyTorch. All results reported below were obtained from a single training run per configuration under this fixed seed; multi-seed training with mean ± standard deviation reporting and statistical significance testing between the compared models is identified as future work in Section 4.

During training, the input image resolution was set to 640×640 pixels. The initial learning rate (lr0) was 0.03, the momentum factor was 0.937, and the batch size was 8. The number of training epochs was 200. Stochastic Gradient Descent (SGD) was used as the optimizer to improve convergence stability. No pre-trained weights were used, and all models were trained from scratch under identical hyperparameter settings to ensure fair comparison. For inference evaluation, the batch size was set to 1 to simulate real-time deployment conditions. All inference experiments were conducted in FP32 precision. Non-Maximum Suppression (NMS) was applied with a confidence threshold of 0.25 and an IoU threshold of 0.45. Frames per second (FPS) was calculated based on single-image inference time on the NVIDIA RTX 4060 GPU.

To comprehensively evaluate the model’s performance in detecting tea bud picking points, this study used the average precision for keypoint (${\mathrm{AP}}_{kp50}$), the F1 score, and the average value of all categories (${\mathrm{mAP}}_{kp50}$) to assess the accuracy of tea bud picking point detection. In addition, we used parameters and giga floating-point operations (GFLOPs) to evaluate the model’s complexity [37]. The real-time performance of the model was measured in frames per second (FPS).

In the picking point location experiment, the pixel Euclidean distance between the predicted picking point and the actual picking point is used as the core evaluation index to quantify the positioning accuracy of the detection results. Let the normalized coordinates of the actual picking point be (x,y), the normalized coordinates of the predicted picking point be $\left(x^{\prime },y^{\prime }\right)$, and the resolution of the original image be w×h=3024×3024 (where *w* represents the image width and *h* represents the image height). Then the pixel distance between the two points in the horizontal direction can be shown as Equation (1), and the pixel distance in the vertical direction can be shown as Equation (2). Based on the horizontal and vertical pixel distances, the pixel Euclidean distance between the two points can be shown as Equation (3):

(1) $$d_{x}=w\cdot \left|x-x^{\prime }\right|$$

(2) $$d_{y}=h\cdot \left|y-y^{\prime }\right|$$

(3) $$d_{e}=\sqrt{d_{x}^{2}+d_{y}^{2}}=\sqrt{[w\cdot |x-x^{\prime }{\left|\right]}^{2}+[h\cdot |y-y^{\prime }{\left|\right]}^{2}}$$

where $d_{x}$ and $d_{y}$ represent the horizontal and vertical pixel deviations, respectively. The smaller the value of $d_{e}$, the smaller the localization deviation between the predicted picking points and the ground-truth picking points, and the higher the localization accuracy of the model.

In addition, to comprehensively evaluate the overall positioning accuracy and performance stability of the model, we added the standard deviation of Euclidean distance to the evaluation index. Let there be *N* tea bud picking targets in the experiment, and let $d_{e,i}$ be the pixel Euclidean distance of the *i*-th target. The average Euclidean distance ${\bar{d}}_{e}$ of all targets can be calculated using Equation (4). The standard deviation of the Euclidean distance can be calculated using Equation (5):

(4) $${\bar{d}}_{e}=\frac{1}{N}\sum_{i=1}^{N}d_{e,i}$$

(5) $$\sigma_{d_{e}}=\sqrt{\frac{1}{N}\sum_{i=1}^{N}{\left(d_{e,i}-{\bar{d}}_{e}\right)}^{2}}$$

where *N* represents the total number of targets. $\sigma_{d_{e}}$ represents the standard deviation of the Euclidean distance. The smaller the value, the smaller the fluctuation of the model’s localization error across different samples, and the more stable its performance; conversely, the larger the value, the greater the difference in the model’s localization performance for some samples.

### 2.2. Experimental Comparison of Different WTConv Module Addition Strategies

**Figure 1:**
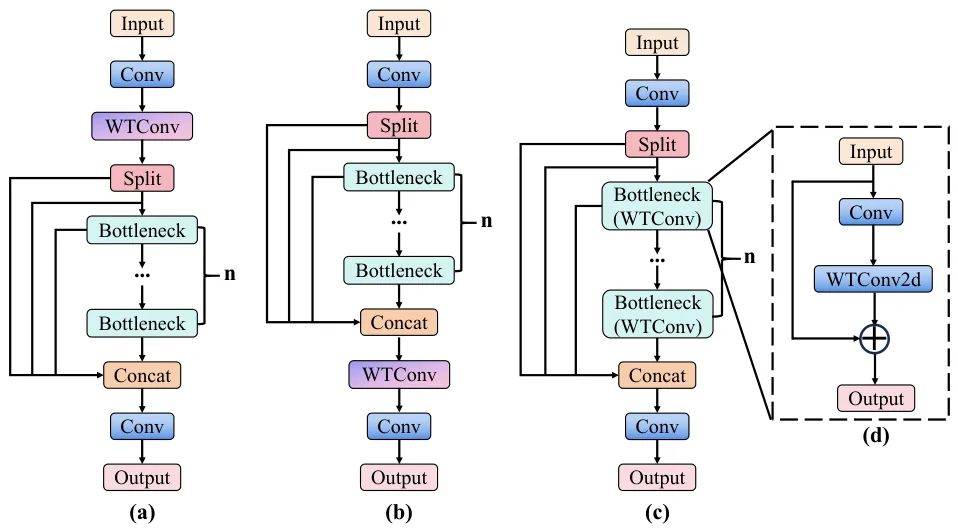
The strategy for adding the WTConv module at different locations within the C2f module. (**a**) Adding it after the cv1 layer. (**b**) Adding it before the cv2 layer. (**c**) Integration within the bottleneck module. (**d**) Internal structure of the modified bottleneck embedded with WTConv2d.

To explore the optimal improvement effect of the WTConv module in the C2f structure, this study designed three improvement strategies, as shown in Figure 1a–c. Strategy 1 (Figure 1a) places it after the first convolutional layer (cv1) to enhance the representation ability of early features; Strategy 2 (Figure 1b) introduces it before the second convolutional layer (cv2) to optimize the extraction process of mid-term features; Strategy 3 (Figure 1c) improves the bottleneck structure to significantly improve the detection performance of the model in the feature processing stage.

**Figure 2:**
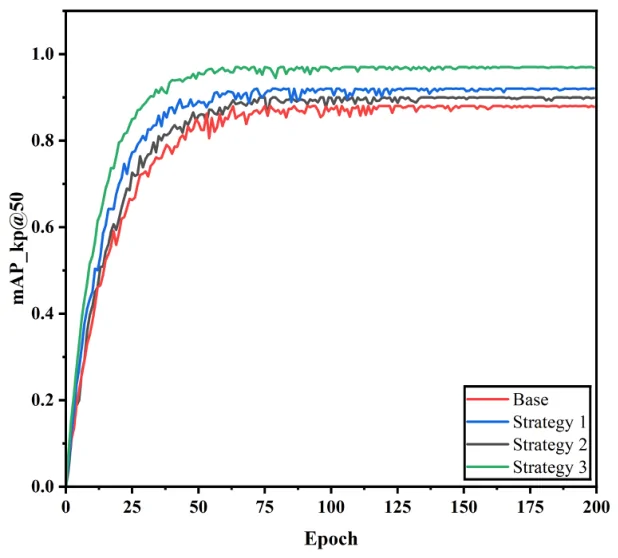
Visual comparison of different WTConv addition strategies on ${\mathrm{mAP}}_{kp50}$.

Compared to the baseline model, all three improvement strategies enhanced detection performance and computational efficiency, as shown in Table 1. Strategy 3 achieved the best performance. Specifically, compared to the baseline model, its ${\mathrm{mAP}}_{kp50}$ and F1 scores improved by 2.28 and 2.87 percentage points, respectively, while the number of parameters and computational complexity decreased by 12.4% and 20.4%, respectively, and its inference speed was also the fastest. It is worth noting that although Strategy 1 outperforms Strategy 2 in overall performance, its ${\mathrm{mAP}}_{kp50}$ and F1 score are still slightly lower than those of Strategy 3. Meanwhile, compared with Strategy 3, Strategy 1 has 11.69% more parameters and 16.81% more GFLOPs, while its inference speed is 19.27% lower, indicating that this strategy comes at a cost in terms of computational efficiency. The performance variations of different strategies on ${\mathrm{mAP}}_{kp50}$ during training are illustrated in Figure 2.

Overall, the superior performance of Strategy 3 is mainly attributed to the structural characteristics of the bottleneck layer in the C2f module. As the core unit of feature transformation, the bottleneck layer plays a crucial role in feature aggregation and residual information fusion. Introducing the WTConv module at this location allows for effective expansion of the multi-scale receptive field in the residual path, enhancing contextual modeling capabilities while maintaining gradient stability. Compared to introducing WTConv into shallow or intermediate layers, embedding it into the bottleneck structure avoids redundant enhancement of lower-level features, thereby reducing unnecessary computational overhead and achieving a better balance between detection accuracy and model lightweighting.

### 2.3. Effectiveness Testing of Different Upsampling Methods

To further evaluate the performance advantages of DySample, this study selected three mainstream upsampling methods, CARAFE [38], Efficient Upsampling Block (EUCB) [39], and Converse2D [40], for comparative experiments. DySample outperforms other mainstream methods in all indicators.

Specifically, its ${\mathrm{mAP}}_{kp50}$ and F1 scores reached 90.18% and 90.04%, respectively, which are 1.92 and 2.63 percentage points higher than the baseline model, respectively. At the same time, its computational cost and real-time performance are also the best. In contrast, EUCB performed poorly, with its detection performance and computational efficiency both below average. It is worth noting that although CARAFE and Converse2D perform better in terms of parameter count and computational cost, their detection performance is significantly reduced, decreasing by 5.76 and 5.02 percentage points, respectively, compared to the DySample module’s ${\mathrm{mAP}}_{kp50}$. These results fully demonstrate that DySample not only achieves significant improvements in model lightweighting but also maintains superior detection performance, achieving an effective balance between model computational efficiency and feature capture capability. Table 2 shows the comparative experimental results of different upsampling methods on multiple performance metrics.

### 2.4. Verifying the Effectiveness of the Weighted Shape-NWD Loss Function

Shape-NWD integrates two complementary components: the Shape-IoU loss and the Normalized Wasserstein Distance (NWD). Shape-IoU introduces shape and scale information into the IoU calculation through aspect-ratio-aware weight coefficients, so that the regression objective adapts to the elongated and variable bounding boxes of tea buds at different growth stages. NWD models the predicted and ground-truth boxes as two-dimensional Gaussian distributions and measures their distance through the Wasserstein metric, which yields stable and meaningful gradients even when the two boxes have little or no overlap—a common situation for small and densely packed tea bud targets. By combining the shape adaptivity of Shape-IoU with the non-overlapping robustness of NWD, the weighted Shape-NWD loss mitigates the gradient vanishing problem of conventional IoU loss and strengthens small-target regression stability, thereby improving the localization accuracy of small and occluded tea buds.

The contribution of the weighted Shape-NWD loss is confirmed by the module ablation study in Table 3: after integrating Shape-NWD on top of the C2f-WTConv and DySample modules, the ${\mathrm{mAP}}_{kp50}$ and F1 score increased by 2.56 and 1.86 percentage points, respectively.

### 2.5. Ablation Experiment

To verify the effectiveness of the proposed improved model in detecting tea bud picking points and to analyze the impact of each improvement on performance, this study conducted ablation experiments on the C2f-WTConv module, Dysample module, and weighted Shape-NWD loss function. The results are shown in Table 3.

The baseline model achieves an ${\mathrm{mAP}}_{kp50}$ of 88.26%, which demonstrates strong detection accuracy, but its computational efficiency leaves room for improvement: the inference speed is 58.47 FPS and the model requires 11.13 M parameters and 28.4 GFLOPs. After WTConv is introduced into the C2f structure of the feature extraction stage, ${\mathrm{mAP}}_{kp50}$ and the F1 score increase by 2.28 and 2.87 percentage points, respectively, while Params and GFLOPs decrease by 12.40% and 20.42%, and the inference speed improves by 31.59%, all relative to the baseline. This suggests that WTConv, by enlarging the effective receptive field through multi-level wavelet decomposition at a small parameter cost, enables the model to capture multi-scale contextual information more flexibly, improving both computational efficiency and the fine-grained detection of picking points.

Introducing DySample to replace the conventional upsampling operator further reduces Params and GFLOPs by 36.51% and 10.18%, respectively, and raises the inference speed by 7.27% relative to the previous configuration, reaching 82.53 FPS. This structural optimization is accompanied by a slight decrease in ${\mathrm{mAP}}_{kp50}$ and the F1 score, by 0.23 and 0.19 percentage points, respectively. This behavior contrasts with Table 2, in which DySample used alone on the baseline improves ${\mathrm{mAP}}_{kp50}$ from 88.26% to 90.18% by replacing fixed interpolation with learnable sampling. When combined with C2f-WTConv, however, the wavelet-domain convolutions already provide enlarged multi-scale contextual information, so the benefit of learnable resampling partially overlaps with what C2f-WTConv has captured, explaining the marginal accuracy change. In exchange, the combination substantially reduces parameters and accelerates inference, indicating that DySample acts primarily as a lightweighting mechanism in the full model: it trims parameters and speeds up inference through learnable sampling-point offsets at the cost of a marginal accuracy drop that is acceptable for resource-constrained edge deployment.

Finally, replacing the bounding box regression loss with the weighted Shape-NWD loss improves ${\mathrm{mAP}}_{kp50}$ and the F1 score by 2.56 and 1.86 percentage points, respectively, while Params and GFLOPs further decrease by 15.67% and 5.91%, and the inference speed increases by 4.03%, all relative to the previous configuration. These results show that Shape-NWD improves the localization accuracy of picking points by dynamically adjusting the shape and scale weights, and that it also enables more efficient feature utilization, further reducing the computational burden. Overall, the combination of the three improvements yields a model with substantially better detection accuracy, computational efficiency, and real-time performance, providing a reliable technical solution for the practical detection of tea bud picking points.

### 2.6. Overall Performance Comparison with Mainstream Detection Models

To comprehensively evaluate the performance of the LTK-YOLO model, this study compared it with five other mainstream keypoint detection models, including YOLOv5s-Pose, YOLOv7s-Pose, YOLOv8s-Pose (baseline), YOLOv11s-Pose, and YOLOv12s-Pose. The experimental results are shown in Table 4.

While maintaining its lightweight advantage, LTK-YOLO achieves the best overall detection performance. Compared with the baseline YOLOv8s-Pose, it improves ${\mathrm{AP}}_{kp50}$ by 3.56 and 4.02 percentage points for kp1-1 and kp1-2, respectively, and the F1 score by 4.54 percentage points (from 87.41% to 91.95%). It also has the lowest Params among all compared models, the second-lowest GFLOPs after YOLOv5s-Pose, and the fastest inference speed, outperforming the other mainstream models overall. These results further demonstrate that, by introducing the lightweight C2f-WTConv module, the adaptive upsampling operator DySample, and the weighted Shape-NWD loss function, LTK-YOLO achieves a favorable balance among detection performance, computational efficiency, and real-time performance, making it particularly suitable for agricultural scenarios with stringent real-time requirements.

Recently, ref. [28] proposed a tea bud pose estimation and grading detection network based on an improved YOLOv7. Since their model and dataset are not publicly available, a direct re-implementation comparison was not possible; we therefore compare the two works qualitatively based on their reported results. Their work targets joint pose estimation and grade classification of tea buds, whereas LTK-YOLO focuses on keypoint-level picking point localization defined by agronomic harvesting standards. With 5.22 M parameters, 19.1 GFLOPs, an inference speed of 85.86 FPS, and an average Euclidean localization error below 15 pixels, LTK-YOLO provides a lightweight solution that is well suited for deployment on resource-constrained tea-picking equipment.

From a deployment perspective, the 85.86 FPS inference speed corresponds to a latency of about 11.6 ms per frame, so visual detection occupies only a small fraction of a robotic picking cycle. In tea-harvesting robotic systems, the cycle time per bud is dominated by manipulator motion and end-effector actuation rather than perception; the latency budget of LTK-YOLO therefore does not constitute a bottleneck for bud-to-bud throughput. Moreover, its reduced parameter count (5.22 M) makes it suitable for edge platforms such as the NVIDIA Jetson series, where the achievable frame rate on embedded hardware, rather than on a desktop GPU, is the relevant constraint for real-time operation.

### 2.7. Analysis of Prediction Results from Different Models

**Figure 3:**
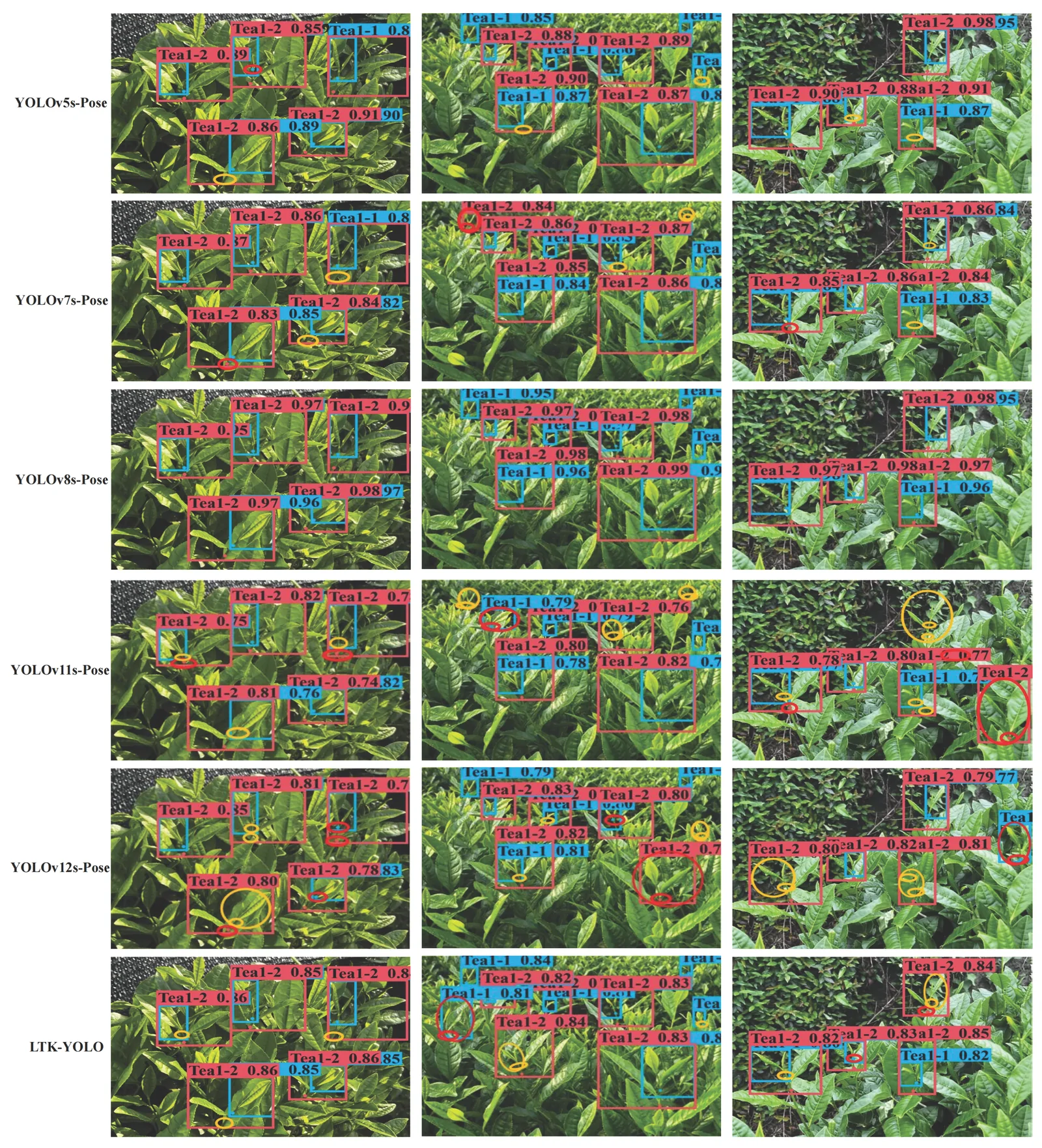
Visualization results of different keypoint detection models in different scenarios.

To intuitively evaluate the performance of the LTK-YOLO model in the picking point detection task, different keypoint detection models were compared on test images under various representative scenarios, as shown in Figure 3. In the figures, red circles denote falsely detected picking points and tea bud targets, while orange circles indicate missed detections. Overall, LTK-YOLO demonstrates superior detection accuracy and robustness, particularly in challenging conditions involving small targets, occlusion, and complex visual interference. Compared with other models, LTK-YOLO effectively reduces both false positives and false negatives.

Specifically, in strong sunlight scenarios, the presence of extensive shadows significantly weakens the visual features of tea buds in shaded regions. YOLOv5s-Pose exhibits the highest number of false positives and false negatives, while other comparative models also show noticeable deviations in picking point localization. In long-distance scenarios, tea buds appear as small targets, making it difficult to accurately distinguish their features and corresponding picking points. YOLOv7s-Pose not only misses multiple picking points but also produces incorrect tea bud grade classifications. Both YOLOv5s-Pose and YOLOv8s-Pose fail to precisely localize the true picking points. In complex background scenarios, the high similarity in color and texture between tea buds and surrounding vegetation significantly increases the difficulty of detection. YOLOv5s-Pose misidentifies independent leaves as one bud and one leaf and exhibits errors in picking point prediction. Other comparative models suffer from high false negative rates, with evident localization deviations.

Overall, LTK-YOLO consistently achieves more accurate and stable detection results across all challenging scenarios, demonstrating its effectiveness and robustness for tea bud picking point detection in natural environments.

In summary, compared to other models that exhibit unstable detection performance in scenarios with occluded or densely distributed targets, and are prone to issues such as missed detections, false detections, and redundant detection boxes due to target overlap and complex backgrounds, the LTK-YOLO model demonstrates significant performance advantages. In various complex tea garden scenarios, this model accurately locates tea bud picking points while maintaining low missed detection and false detection rates, fully demonstrating its excellent environmental adaptability and detection robustness. This not only provides high-precision guidance information for automated tea-picking robots but also lays a solid technical foundation for achieving continuous, reliable, all-weather operation.

### 2.8. Comparative Experiment on the Location of Tea Bud Picking Points

To verify the effectiveness of LTK-YOLO in the tea bud picking location task, this study compared its performance with five other mainstream keypoint detection models. All 400 images of the test set (Table 5) were used for evaluation. For each model, 3296 tea bud picking points that were successfully detected and matched to the annotated ground truth, including 1872 kp1-1 and 1424 kp1-2, were used for the localization error computation. The coordinates of these picking points were used to calculate the average error. Table 6 presents the statistical results of the average localization errors of different models in the X-axis direction, Y-axis direction, and Euclidean distance.

**Figure 4:**
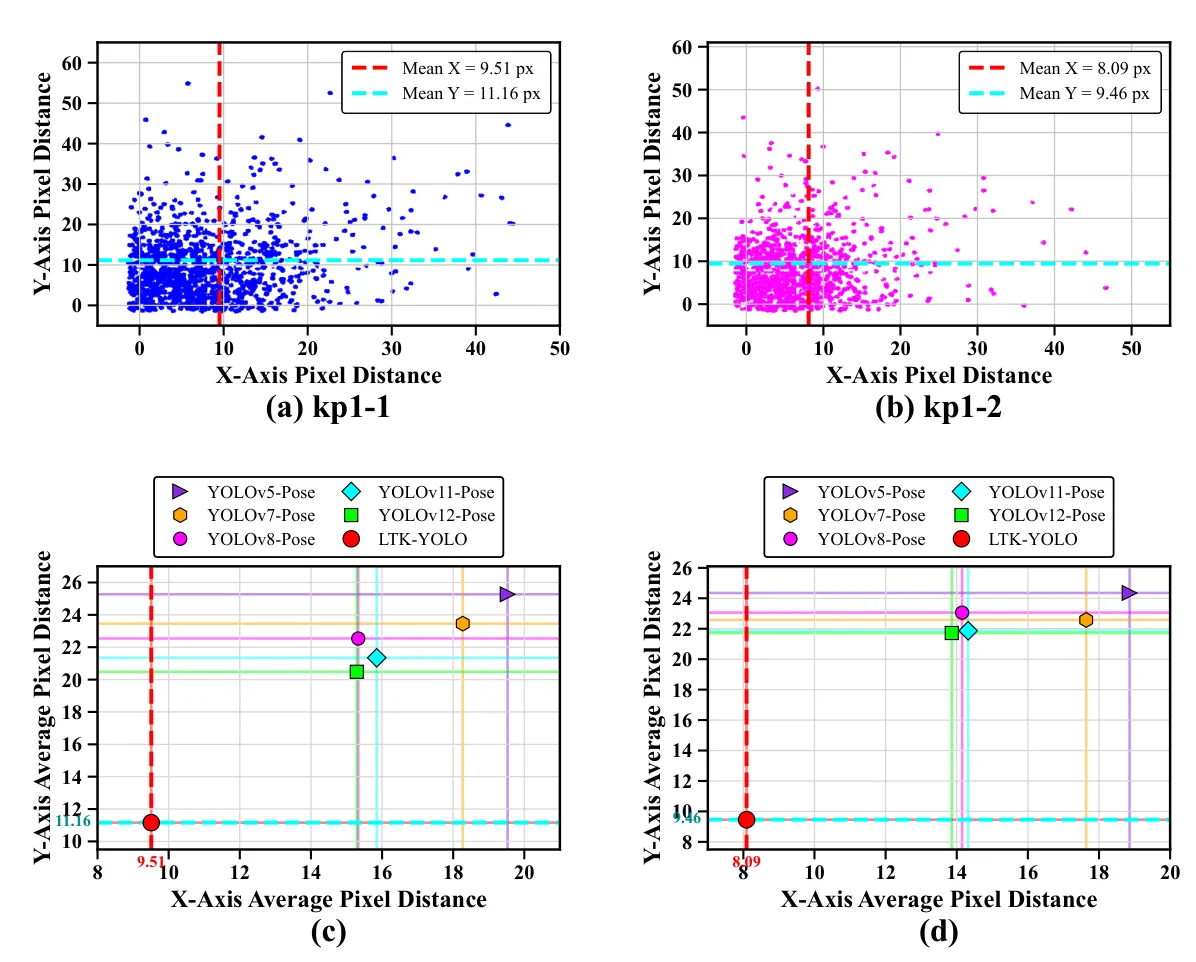
Localization error analysis of tea bud picking points: (**a**,**b**) scatter distribution of the X- and Y-axis errors between the predicted and ground-truth picking points of LTK-YOLO, where the dashed lines indicate the corresponding mean errors; (**c**,**d**) comparison of the average X- and Y-axis localization errors among different models. (**a**,**c**) kp1-1; (**b**,**d**) kp1-2.

Figure 4a,b show the scatter plot distribution of errors between predicted and actual picking points using LTK-YOLO. The X and Y axes represent the horizontal and vertical distance errors, respectively. Furthermore, we use red dashed vertical lines to indicate the average horizontal distance error and cyan dashed horizontal lines to indicate the average vertical distance error. Similarly, Figure 4c,d use different colored horizontal and vertical lines to compare the average distance errors between all predicted and actual points along the X and Y axes for different models.

According to the results in Table 6, the LTK-YOLO model outperforms other mainstream comparative models in terms of average Euclidean distance on both the X and Y axes, indicating its high accuracy in locating picking points. Specifically, its average Euclidean distances at the two picking points are 14.66 pixels and 12.45 pixels, respectively, with standard deviations of 11.03 and 10.17, respectively, both the lowest among all models, indicating its relatively stable performance and low fluctuation in picking point location.

These results demonstrate that LTK-YOLO achieves both the lowest average localization error and the smallest error fluctuation among all compared models, indicating that it can provide stable and accurate picking point localization that meets the practical requirements of tea-picking equipment.

## 3. Materials and Methods

### 3.1. Data Acquisition

**Figure 5:**
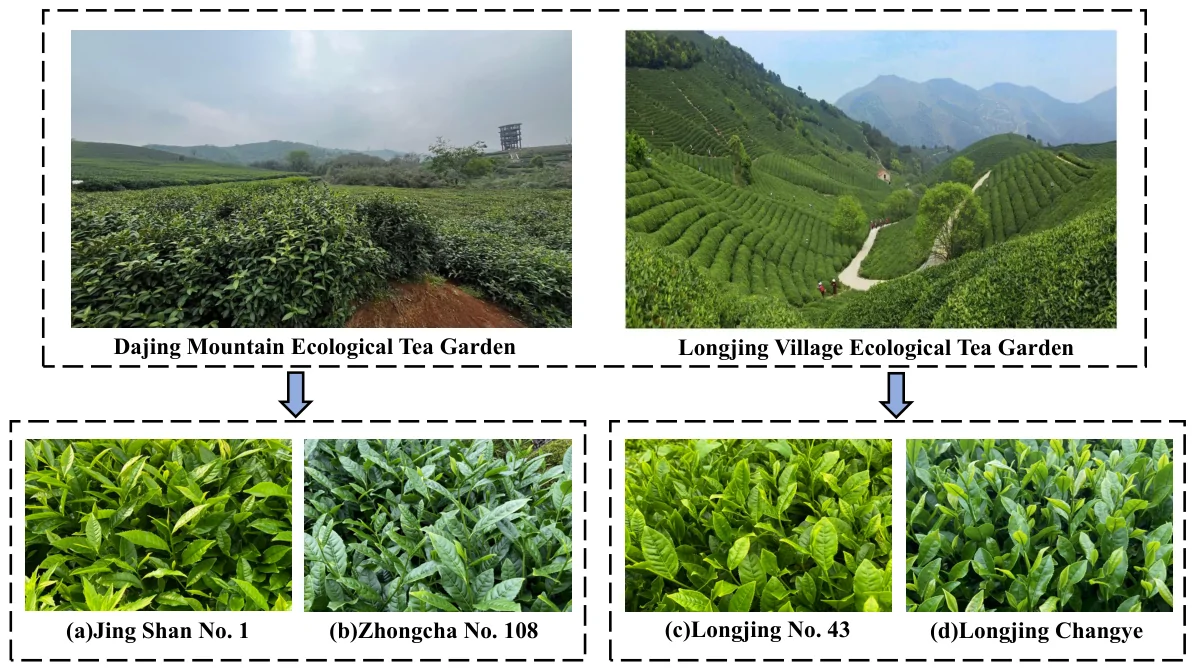
Location and variety of tea bud image dataset collected.

This study used a Canon EOS 90D digital camera equipped with an EF 50 mm f/1.8 STM lens for image acquisition. Data collection focused on four representative high-quality tea bud varieties (“Jingshan No. 1”, “Zhongcha No. 108”, “Longjing 43”, and “Longjing Changye”). From 1 March to 1 May 2024, images were collected at the Dajingshan Ecological Tea Garden (Yuhang District) and the Longjing Village Tea Garden (Xihu District), Hangzhou, Zhejiang Province. The final dataset consists of 4000 original images, with 1000 images per variety. Figure 5 illustrates the collection locations and representative samples.

**Figure 6:**
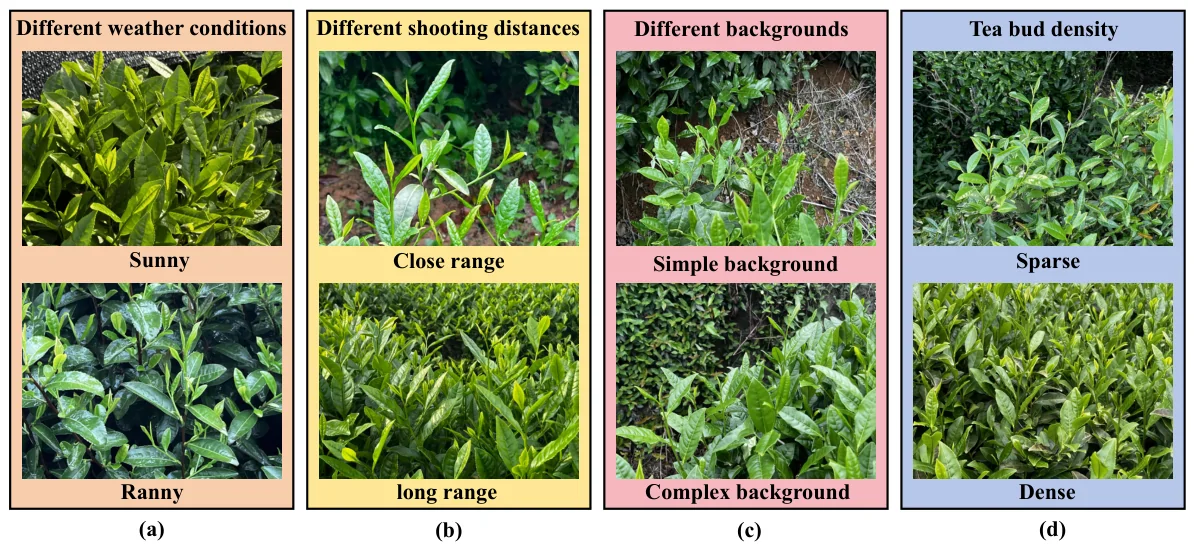
Images of tea buds in different scenarios. (**a**) Different weather conditions. (**b**)Different shooting distances. (**c**) Different backgrounds. (**d**)Tea bud density.

To simulate realistic field operation conditions, variations in target distance, viewing angle, background complexity, and illumination were deliberately considered. The camera-to-target distance ranged from 30–90 cm, and images were captured from multiple perspectives. Weather conditions during acquisition included approximately 60% sunny, 30% cloudy, and 10% lightly rainy conditions, ensuring diversity in lighting scenarios. All images were captured at a resolution of 3024 × 3024 pixels and stored in JPEG format. Images from both tea gardens were randomly mixed before dataset splitting to ensure diversity across training, validation, and test sets. This strategy improves the robustness of the model evaluation under cross-location variations. Representative images under different environmental conditions are shown in Figure 6.

### 3.2. Data Preprocessing

**Figure 7:**
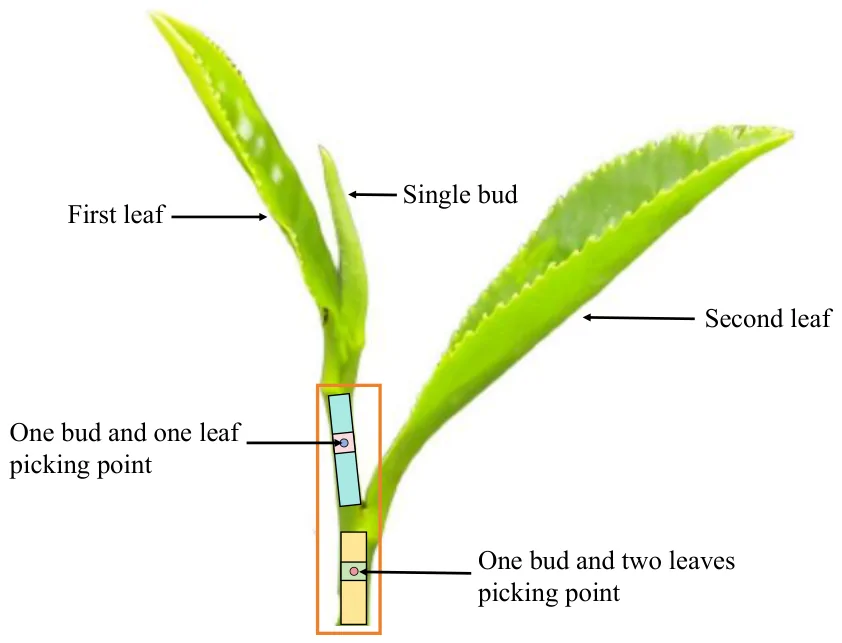
Schematic diagram of tea bud picking area.

Considering that premium tea products are typically harvested as “one bud and one leaf” and “one bud and two leaves”, the picking points corresponding to these two grades were defined as the detection targets. The open-source annotation tool LabelImg was used to annotate bounding boxes and keypoints. Unlike fixed geometric corners, tea bud picking points do not correspond to visually salient features but must lie within a suitable agronomic picking range. According to the national standard GB/T 18650 for Longjing tea [41], which defines the tender shoot grades of premium green tea, the optimal picking area was defined as the stem segment between the leaf base node and the stem connection node, specifically 1/3 to 1/2 of its length. The picking points for “one bud and one leaf” and “one bud and two leaves” were manually marked within this defined region. The former was labeled kp1-1, and the latter kp1-2. Importantly, each tea bud instance contains only one keypoint label (either kp1-1 or kp1-2). The tea bud picking area is shown in Figure 7.

**Figure 8:**
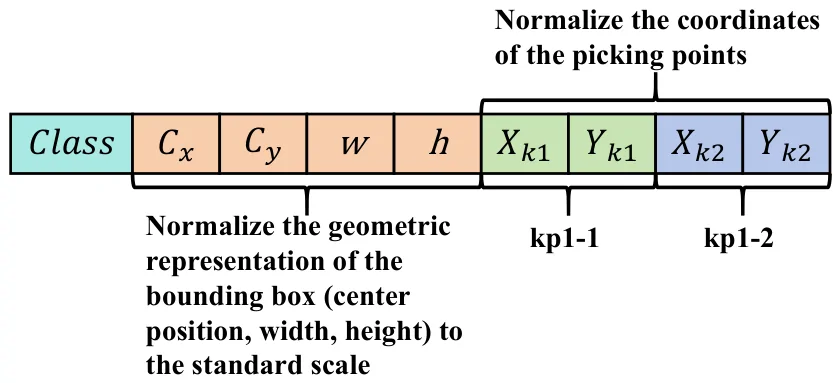
Labeling information for tea buds and picking points.

To ensure annotation consistency, all labeling work was completed by the same trained researcher over a 12-week period. Because the picking point is defined geometrically by the national standard (a fixed fraction of the stem segment) rather than by a salient visual landmark, keypoint placement is largely rule-based, which limits the subjective variability expected between annotators. For quality assurance, a secondary manual review was conducted on randomly selected samples to verify annotation consistency. In cases of partial occlusion, keypoints were labeled according to the estimated stem position within the defined picking region, ensuring annotation stability under occlusion scenarios. After annotation, bounding box and keypoint coordinates were normalized by dividing each component by the image width and height, ensuring all coordinate values were within the range [0,1]. The normalized labels were stored in TXT format, with each line corresponding to one annotated instance, as shown in Figure 8.

### 3.3. Dataset Details

To ensure objective model evaluation and statistical validity, the dataset was divided into training, validation, and test sets at a ratio of 8:1:1 using a stratified random split, stratified by tea variety and tea garden, so that each subset preserves the original proportions of varieties and collection locations. The distribution of keypoint categories is as follows: kp1-1 accounts for approximately 51.08% of the dataset, while kp1-2 accounts for 48.92%, reflecting the natural proportion of harvesting grades in field conditions. Detailed dataset statistics are summarized in Table 5.

During training, different background environments may lead to interference in the identification of tea bud features. To address this issue, online data augmentation methods such as random image scaling and mosaic data augmentation were employed during training. Finally, all images were resized to 640 × 640 pixels to balance training efficiency and detection accuracy.

### 3.4. Improved LTK-YOLO Model

While YOLOv8s-Pose performs well on general keypoint detection tasks, it falls short in unstructured and complex tea garden scenarios, particularly in handling the localization and detection of picking points with dense occlusion, different varieties, varying lighting conditions, and multi-scale targets. Furthermore, the baseline model suffers from complex parameters and computational overhead, increasing the difficulty of deployment on resource-constrained devices.

**Figure 9:**
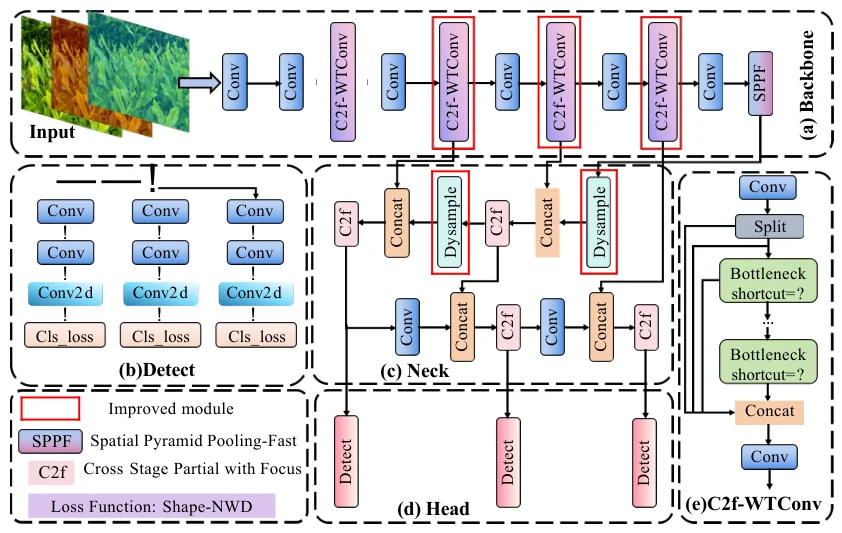
Overall structure of LTK-YOLO.

To address these issues, LTK-YOLO makes key improvements to YOLOv8s-Pose to significantly enhance model performance in picking tasks. Specifically, we introduce the WTConv module into the C2f module of the backbone network. By performing wavelet domain convolutions, we enhance the model’s ability to accurately identify the contours and shapes of tea bud targets, thereby capturing broader contextual information while reducing the number of parameters. In addition, we introduce a lightweight and efficient dynamic upsampling module, DySample, in the feature fusion stage. It adaptively learns the sampling point offset to achieve precise alignment of small targets and preservation of fine-grained edge details, demonstrating excellent performance in dense tea bud detection and localization tasks. Finally, the weighted Shape-NWD loss function optimizes the bounding box regression accuracy obtained in the target detection task by adjusting the weights to address the differences in the shape and size of the tea buds, significantly enhancing the model’s ability to locate tea bud picking points. The overall structure of the LTK-YOLO model is shown in Figure 9.

#### 3.4.1. Lightweight C2f-WTConv Module

**Figure 10:**
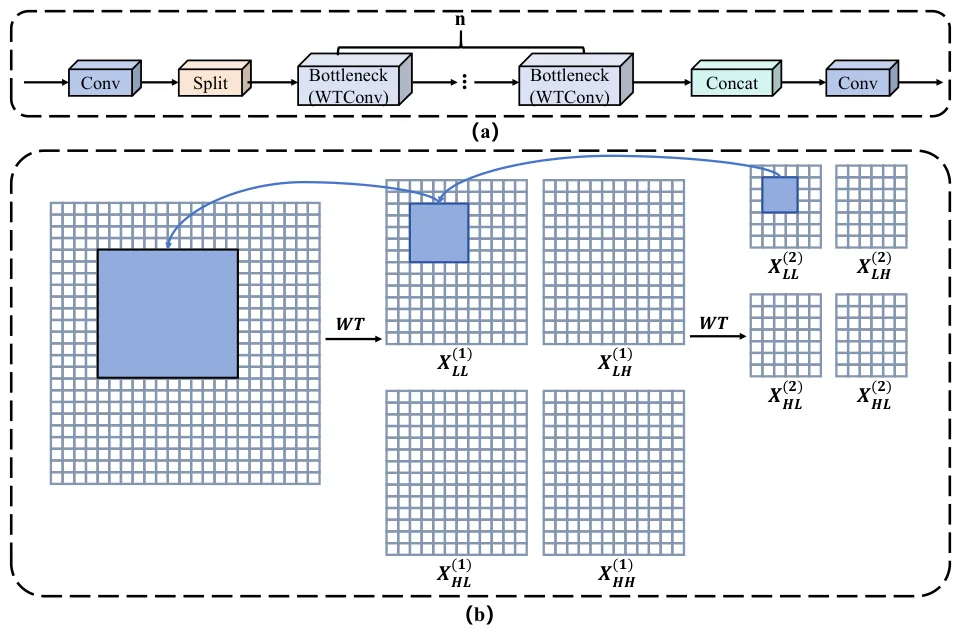
Overall structure of the proposed C2f-WTConv module and the internal principle of WTConv. (**a**) C2f-WTConv module. (**b**) The implementation principle of the WTConv module.

The C2f-WTConv module is designed to enlarge the effective receptive field while maintaining low parameter complexity. In conventional convolutional networks, expanding the receptive field typically relies on increasing kernel size or stacking deeper layers, both of which substantially increase computational cost and parameter redundancy. Such strategies are unfavorable for lightweight deployment in tea-picking robotic systems. To address these issues, this study incorporates the wavelet transform convolution (WTConv) module [42] in the feature extraction stage of the model to effectively improve the bottleneck structure in C2f, forming the C2f-WTConv module, as shown in Figure 10a.

Specifically, wavelet transform can decompose the input features into sub-band signals of different scales, separating the low-frequency component $X_{LL}$ and the high-frequency components $\left(X_{LH},X_{HL},X_{HH}\right)$, and then performing a 3×3 convolution operation on the low-frequency sub-band of $X_{LL}$ in the second-order wavelet domain. Since the two-level Haar decomposition downsamples the low-frequency sub-band by a factor of 4 in each spatial dimension, a single 3×3 convolution on it covers an equivalent region of (3×4)×(3×4)=12×12 at the input resolution, while introducing only the 9 trainable weights of the kernel (per input–output channel pair); the Haar filters themselves are fixed and non-trainable [42]. Afterwards, the output features are obtained by combining the components for reconstruction and performing inverse wavelet transform. This method enables the model to simultaneously capture the local key details and global structural information of the image, which not only improves the computational efficiency but also significantly enhances the model’s ability to identify tea bud picking points. The overall wavelet decomposition process formula is as follows:

(6) $$X_{LL},X_{LH},X_{HL},X_{HH}=WT\left(X\right)$$

where WT(·) represents the wavelet transform function, used to perform multi-scale decomposition of input features. $X_{LL}$ represents the low-frequency component. The high-frequency components are $X_{LH}$, $X_{HL}$, and $X_{HH}$, which are used to extract edge features sequentially along the horizontal, vertical, and diagonal axes. For the low-frequency component, it is processed layer by layer through a multi-level iterative decomposition strategy to achieve a gradual expansion of the receptive field. The formula is expressed as follows:

(7) $$X_{LL}^{\left(i\right)},X_{H}^{\left(i\right)}=WT\left(X_{LL}^{\left(i-1\right)}\right)$$

where $X_{LL}^{\left(i-1\right)}$ represents the low-frequency component from the previous stage, which is further decomposed by wavelet transform to obtain the low-frequency component $X_{LL}^{\left(i\right)}$ and high-frequency component $X_{H}^{\left(i\right)}$ of the *i*-th layer. Subsequently, a convolution operation is performed on them to enhance feature representation, as shown in the following formula:

(8) $$Y_{LL}^{\left(i\right)},Y_{H}^{\left(i\right)}=Conv\left(W^{\left(i\right)},\left[X_{LL}^{\left(i\right)},X_{H}^{\left(i\right)}\right]\right)$$

where $W^{\left(i\right)}$ represents the kernel function, used to perform efficient convolution operations on the low-frequency component $X_{LL}^{\left(i\right)}$ and the high-frequency component $X_{H}^{\left(i\right)}$, resulting in the updated low-frequency and high-frequency components $Y_{LL}^{\left(i\right)}$ and $Y_{H}^{\left(i\right)}$ of the *i*-th layer, respectively. Finally, an inverse wavelet transform is performed:

(9) $$Z^{\left(i\right)}=IWT\left(Y_{LL}^{\left(i\right)}+Z^{\left(i+1\right)},Y_{H}^{\left(i\right)}\right)$$

where IWT(·) represents the inverse wavelet transform function, which is used to reconstruct and fuse the convolutional features to generate the fused feature map $Z^{\left(i\right)}$ for the current stage.

From an implementation perspective, WTConv is implemented using PyTorch tensor operations combined with fixed Haar filter kernels, without introducing additional trainable wavelet parameters. Therefore, the module preserves compatibility with standard GPU acceleration and does not significantly increase parameter count or computational burden.

#### 3.4.2. DySample Adaptive Upsampling Module

**Figure 11:**
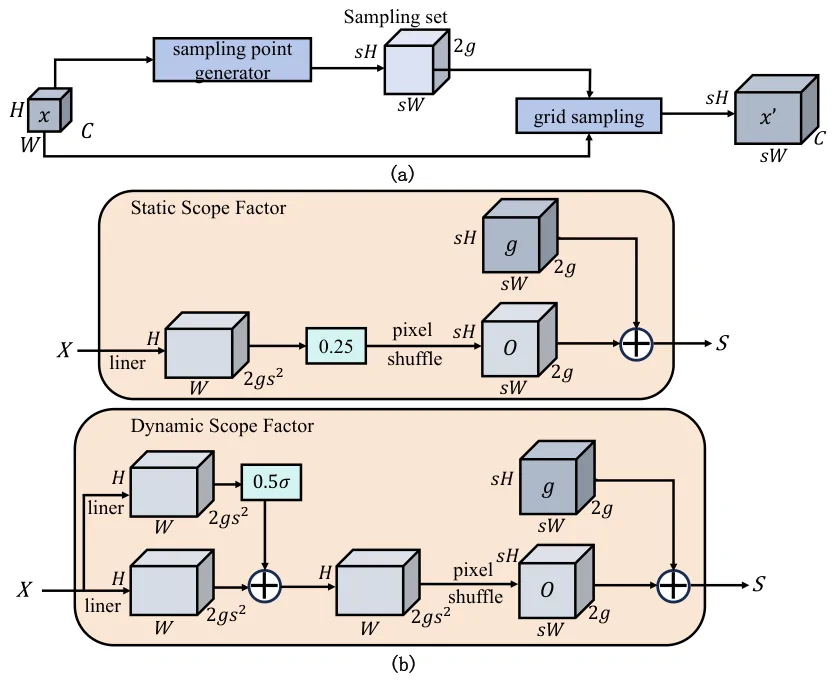
DySample dynamic upsampling module. (**a**) Overall module structure. (**b**) Sampling point generator.

Conventional upsampling operators such as nearest-neighbor or bilinear interpolation rely on fixed sampling grids and therefore cannot adapt to spatial variations in feature distributions. This limitation is particularly critical for small tea bud targets under occlusion and complex backgrounds. To overcome these limitations, we adopt the DySample module [43] in the feature fusion stage of the model to effectively improve the traditional upsampling operator. It replaces fixed interpolation with a learnable sampling mechanism that dynamically adjusts sampling locations according to input features while maintaining low computational overhead. Figure 11 illustrates the dynamic upsampling architecture of DySample.

Given an input feature map $X\in R^{C\times H\times W}$, DySample first learns pixel-wise adaptive sampling offsets through lightweight linear projections. Let *s* denote the upsampling scale factor (e.g., s=2 for 2× upsampling). A linear projection layer expands the channel dimension to $2s^{2}$, enabling the prediction of horizontal and vertical displacements for each upsampled location. The predicted offsets are normalized using a Sigmoid function and scaled to constrain the displacement range, ensuring spatial stability during sampling. The offset tensor is computed as:

(10) $$O=0.5\times sigmoid\left(linear_{1}\left(X\right)\right)\times linear_{2}\left(X\right)$$

where the scaling factor 0.5 limits excessive displacement and preserves local structural consistency. The two linear layers serve complementary roles: the first generates the spatial offset distribution, while the second modulates feature intensity to refine the magnitude of displacement, thereby enhancing the coupling between semantic features and spatial alignment.

Let $G\in R^{2\times sH\times sW}$ denote the regular sampling grid corresponding to standard upsampling. The learned offset tensor *O* is reshaped to match the spatial resolution of the upsampled grid and added to the regular grid to obtain the adaptive sampling set:

(11) S=G+O

Finally, differentiable bilinear interpolation is performed based on the updated sampling grid using the PyTorch function:

(12) $$X^{\prime }=grid\_sample\left(X,S\right)$$

where $X^{\prime }\in R^{C\times sH\times sW}$ denotes the upsampled feature map.

Compared with dynamic convolution-based upsampling operators that require generating spatially varying convolution kernels, DySample directly learns sampling coordinates without introducing additional heavy subnetworks. This design reduces parameter overhead and computational complexity while improving spatial alignment and detail preservation, making it well suited for lightweight real-time deployment in tea-picking robotic systems.

#### 3.4.3. Weighted Shape-NWD Loss Function

Accurate bounding box regression is crucial for keypoint localization tasks. The traditional IoU loss has gradient vanishing problems when the predicted box and the ground-truth box do not overlap, and it does not consider the shape and scale differences of the targets. For tea buds of different varieties and growth stages, their bounding box shapes vary greatly, so a more robust loss function is needed.

The Shape-IoU loss [44] introduces shape information into the IoU calculation by constructing shape weight coefficients, and the Normalized Gaussian Wasserstein Distance (NWD) [45] models the distance between bounding boxes for small targets. On this basis, this study further integrates these two components to construct the weighted Shape-NWD loss function, which improves the regression accuracy and stability of small targets.

First, the shape weight coefficient is defined based on the aspect ratio of the ground-truth box:

(13) $$ww=\frac{2\times {\left(w^{gt}\right)}^{2}}{{\left(w^{gt}\right)}^{2}+{\left(h^{gt}\right)}^{2}+scale}$$

(14) $$hh=\frac{2\times {\left(h^{gt}\right)}^{2}}{{\left(w^{gt}\right)}^{2}+{\left(h^{gt}\right)}^{2}+scale}$$

where $w^{gt}$ and $h^{gt}$ represent the width and height of the ground truth bounding box, respectively. The scaling factor scale is determined according to the statistical distribution of ground-truth bounding box sizes in the training dataset. Specifically, it is derived from the average width-height scale of annotated targets to normalize size variation across different tea bud instances, ensuring stable weight coefficients under multi-scale conditions. Then, the distance loss $distance_{shape}$ is calculated using the defined horizontal and vertical weight coefficients.

(15) $$distance_{shape}=hh\times \frac{{\left(x_{c}-x_{gt}\right)}^{2}}{c^{2}}+ww\times \frac{{\left(y_{c}-y_{gt}\right)}^{2}}{c^{2}}$$

where $\left(x_{c},y_{c}\right)$ and $\left(x_{gt},y_{gt}\right)$ represent the center coordinates of the predicted bounding box and the ground truth bounding box, respectively. *c* represents the diagonal distance of the smallest bounding rectangle containing these two boxes. This distance term incorporates a shape weight coefficient in the calculation, aiming to quantify the spatial deviation between the two center points after shape adaptive adjustment. This further defines the formula for calculating the shape loss:

(16) $$\Omega_{shape}=\sum_{t=w,h}{\left(1-e^{-w_{t}}\right)}^{\theta },\;\theta =4$$

(17) $$w_{w}=hh\cdot \frac{|w-w^{gt}|}{\max(w,w^{gt})}$$

(18) $$w_{h}=ww\cdot \frac{|h-h^{gt}|}{\max(h,h^{gt})}$$

where *w* and *h* represent the width and height of the predicted bounding box, respectively, and t=w,h represents the sum of the losses calculated on *w* and *h* respectively. θ represents the penalty coefficient, used to adjust the sensitivity of the loss. The penalty coefficient θ is set to 4 based on empirical validation. Smaller values reduce sensitivity to shape deviation, whereas excessively large values lead to unstable gradients. The selected value provides a balance between regression sensitivity and training stability. Subsequently, the bounding box position and scale features are fused to obtain the comprehensive distance metric *D*:

(19) $$D=\sqrt{hh{\left(x_{c}-x_{c}^{gt}\right)}^{2}+ww{\left(y_{c}-y_{c}^{gt}\right)}^{2}+\frac{{\left(w-w^{gt}\right)}^{2}+{\left(h-h^{gt}\right)}^{2}}{weight^{2}}}$$

where weight represents the weighting coefficient, fixed at 2, used to balance the contributions of position and scale features in the distance calculation. Finally, the mathematical expression for the Shape-NWD bounding box regression loss after normalization is as follows:

(20) $$NWD^{shape}=e^{-\frac{D}{C}}$$

(21) $$L_{shape-NWD}=1-NWD^{shape}$$

where *C* represents the normalization parameter. It is chosen based on the spatial characteristics of the dataset (including differences in target size and location distribution). The selected value ensures the numerical stability of the exponential term and prevents gradient vanishing or exploding during training.

During training, the overall optimization objective of the proposed model is defined as:

(22) $$L_{total}=L_{det}+\lambda_{kp}L_{kp}+\lambda_{box}L_{Shape-NWD}$$

where $L_{det}$ denotes the detection loss, $L_{kp}$ represents the keypoint regression loss, and $L_{Shape-NWD}$ is the proposed bounding box regression loss. The weighting coefficients $\lambda_{kp}$ and $\lambda_{box}$ are determined empirically to maintain stable multi-task learning and balanced gradient contributions among the objectives. Because evaluating each candidate configuration requires a complete retraining pass, a systematic sensitivity analysis over these hyperparameters (e.g., the penalty coefficient θ and the weight ratios) was not performed in this study and is planned as future work.

## 4. Conclusions

This study aims to address the challenges of accurate and lightweight tea bud picking point localization in unstructured tea garden environments. To achieve this objective, a keypoint-based lightweight detection model, termed LTK-YOLO, was developed based on the YOLOv8s-Pose framework. A dedicated tea bud dataset containing 4000 images with diverse varieties, lighting conditions, occlusion patterns, and scale variations was constructed to support model training and evaluation. By integrating the C2f-WTConv module, the DySample operator, and the weighted Shape-NWD loss function, the proposed model effectively enhances feature representation and regression stability while significantly reducing computational redundancy. Experimental results demonstrate that LTK-YOLO improves ${\mathrm{mAP}}_{kp50}$ from 88.26% to 92.87%, with ${\mathrm{AP}}_{kp50}$ reaching 91.41% and 92.05% for kp1-1 and kp1-2, respectively. The F1 score approaches 92%, and the average Euclidean localization error remains below 15 pixels. Compared with YOLOv8s-Pose, the proposed model reduces parameters and GFLOPs by 53.1% and 32.7%, respectively, while increasing inference speed by 46.8%, demonstrating an effective balance between accuracy and lightweight design.

Despite these promising results, several limitations remain. First, the dataset was collected from tea gardens within a single geographic region and season, which may limit the model’s generalization to other cultivation environments or climatic conditions. Based on the acquisition design, the model is expected to generalize to tea gardens with comparable varieties, canopy density, and illumination and occlusion conditions; deployment in other climatic regions, in other seasons, or on unseen tea varieties is expected to require fine-tuning with locally collected data. Future validation in different tea garden conditions, including multi-regional and multi-season campaigns, is therefore a necessary next step. Second, the current validation is based solely on image-level experiments without deployment on a physical robotic picking system, and annotation consistency relies on a single trained annotator supported by a secondary review rather than on a formal inter-annotator agreement study. Future work will focus on expanding the dataset to multi-regional and multi-season scenarios, quantifying inter-annotator agreement on a relabeled subset, and deploying LTK-YOLO on embedded platforms such as NVIDIA Jetson AGX Orin to evaluate real-world picking performance and system stability. By translating the agronomic picking standards of premium teas into a learnable keypoint localization task and coupling it with a lightweight model deployable on edge hardware, LTK-YOLO provides a practical and cost-effective solution for real-time tea bud picking point detection, offering significant potential to accelerate the intelligent transformation of tea harvesting and reduce labor dependency in large-scale tea production.

## Figures and Tables

**Table 1:** Comparison of detection performance and efficiency between different WTConv improvement strategies and the baseline model.

Method	${\mathrm{mAP}}_{\mathrm{kp}50}$	F1-Score	Params (M)	GFLOPs (G)	FPS
Base	88.26	87.41	11.13	28.4	58.47
Strategy 1	89.97	89.83	10.89	26.4	62.11
Strategy 2	89.02	88.35	10.91	27.2	60.86
Strategy 3	90.54	90.28	9.75	22.6	76.94

**Table 2:** Comparison of detection performance and computational efficiency of different upsampling operators.

Model	Model Size (MB)	GFLOPs	Params (M)	${\mathrm{AP}}_{\mathrm{kp}50}$ (%)		${\mathrm{mAP}}_{\mathrm{kp}50}$ (%)	F1-Score
				kp1-1	kp1-2		
Base	22.42	28.4	11.13	86.17	89.35	88.26	87.41
CARAFE	18.64	21.6	7.95	81.13	82.04	84.42	82.96
EUCB	21.97	27.1	10.28	82.49	84.57	83.79	81.37
Converse2D	20.29	25.3	9.92	83.72	85.35	85.16	85.71
DySample	19.04	23.2	8.36	89.83	91.13	90.18	90.04

**Table 3:** Ablation study on the effectiveness of different improvement modules.

C2f-WTConv	DySample	Shape-NWD	${\mathrm{mAP}}_{\mathrm{kp}50}$ (%)	F1-Score (%)	Params (M)	GFLOPs	FPS
–	–	–	88.26	87.41	11.13	28.4	58.47
✓	–	–	90.54	90.28	9.75	22.6	76.94
✓	✓	–	90.31	90.09	6.19	20.3	82.53
✓	✓	✓	92.87	91.95	5.22	19.1	85.86

**Table 4:** Comparison of detection performance and efficiency between different models.

Model	${\mathrm{AP}}_{\mathrm{kp}50}$ (%)		${\mathrm{mAP}}_{\mathrm{kp}50}$ (%)	F1 (%)	GFLOPs	Params (M)	FPS	Size (MB)
	**kp1-1**	**kp1-2**						
YOLOv5s-Pose	84.58	85.74	85.91	85.37	16.5	7.07	62.18	15.8
YOLOv7s-Pose	87.09	87.18	87.94	87.52	105.1	37.2	26.38	102.3
YOLOv8s-Pose	87.85	88.03	88.26	87.41	28.4	11.13	58.47	27.1
YOLOv11s-Pose	87.53	88.41	89.72	89.45	23.1	9.92	74.92	21.8
YOLOv12s-Pose	87.20	88.26	89.19	88.63	21.4	9.36	79.58	20.7
LTK-YOLO	91.41	92.05	92.87	91.95	19.1	5.22	85.86	18.3

**Table 5:** Statistics of the Tea Bud Keypoint Detection Dataset.

Dataset	Images	kp1-1	kp1-2	Total Targets	kp1-1 Ratio (%)	kp1-2 Ratio (%)
Training set	3200	23,162	22,673	45,835	50.54	49.46
Test set	400	3186	2845	6031	53.64	46.36
Validation set	400	3261	2819	6080	52.85	47.15
Total	4000	29,609	28,337	57,946	51.08	48.92

**Table 6:** Comparison of locations for kp1-1 and kp1-2.

Model	Points	Average Distance (Pixel)			Standard Deviation (Pixel)
		X-Axis	Y-Axis	Euclidean Distance	
YOLOv5s-Pose	kp1-1	19.53	25.27	31.87	26.59
	kp1-2	18.86	24.35	30.82	25.73
YOLOv7s-Pose	kp1-1	18.27	23.46	29.74	25.16
	kp1-2	17.64	22.58	28.69	24.32
YOLOv8s-Pose(base)	kp1-1	15.33	22.54	27.19	23.02
	kp1-2	14.15	23.06	26.91	22.76
YOLOv11s-Pose	kp1-1	15.29	20.48	25.56	21.27
	kp1-2	13.86	21.72	25.76	21.14
YOLOv12s-Pose	kp1-1	15.85	21.35	26.59	22.15
	kp1-2	14.32	21.87	25.93	21.58
LTK-YOLO	kp1-1	9.51	11.16	14.66	11.03
	kp1-2	8.09	9.46	12.45	10.17

## Data Availability

The data, code, and trained weights presented in this study are associated with an ongoing patent application and cannot be publicly released before the intellectual property process is concluded. Until then, they are available from the corresponding author upon reasonable request, and a public release is planned once this process is completed.
